# Extracellular vesicles as mediators of stress response in embryo-maternal communication

**DOI:** 10.3389/fcell.2024.1440849

**Published:** 2024-08-05

**Authors:** Seyed Omid Mousavi, Qurat Ul Ain Reshi, Kasun Godakumara, Suranga Kodithuwakku, Alireza Fazeli

**Affiliations:** ^1^ Institute of Veterinary Medicine and Animal Sciences, Estonian University of Life Sciences, Tartu, Estonia; ^2^ Department of Pathophysiology, Institute of Biomedicine and Translational Medicine, Faculty of Medicine, University of Tartu, Tartu, Estonia; ^3^ Department of Animal Science, Faculty of Agriculture, University of Peradeniya, Peradeniya, Sri Lanka; ^4^ Division of Clinical Medicine, School of Medicine and Population Health, University of Sheffield, Sheffield, United Kingdom

**Keywords:** extracellular vesicles, stress, trophoblast, endometrial cells, miRNA

## Abstract

**Introduction:** The pivotal role of extracellular vesicles (EVs) in facilitating effective communication between the embryo and maternal cells during the preimplantation stage of pregnancy has been extensively explored. Nonetheless, inquiries persist regarding the alterations in EV cargo from endometrial cells under stress conditions and its potential to elicit specific stress responses in trophoblast cells. Thus, the aim of this study was to elucidate the involvement of EV miRNA miRNAs in transmitting stress signals from maternal cells to trophoblasts.

**Methods:** The receptive endometrial epithelium analogue RL95-2 cells were subjected to stress induction with 200 µM CoCl_2_ for 24 h before EV isolation. JAr trophoblast spheroids, which serve as embryos, were subjected to treatment with stressed or unstressed EVs derived from RL95-2 cells for 24 h. Transcriptomic alterations in the treated JAr spheroids as well as in the untreated group, as a negative control, were investigated by mRNA sequencing. Furthermore, the changes in EV miRNAs were assessed by sequencing EV samples.

**Results:** A comprehensive analysis comparing the miRNA profiles between stressed and unstressed EVs revealed significant changes in 25 miRNAs. Furthermore, transcriptomic analysis of JAr spheroids treated with stressed RL95-2EVs versus unstressed EVs or the untreated group demonstrated 6 and 27 differentially expressed genes, respectively. Pathway enrichment analysis showed that stressed EVs induce alterations in gene expression in trophoblast cells, which is partially mediated by EV microRNAs.

**Discussion:** Our results suggest that EVs can transfer stress signals from endometrial cells to the embryo. These discoveries shed new light on the mechanism underlying implantation failures under stress conditions. Unraveling the role of EVs in transmitting stress signals, can extend our knowledge to pave the way for targeted interventions to manage stress-related implantation failures.

## 1 Introduction

Human embryo implantation is a critical step in the process of human reproduction leading to placenta formation and foetal development. The main steps of this intricate process include embryo apposition, attachment to the endometrium, and invasion, which require the active participation of both the embryo and the endometrium (Kim and Kim, 2017; Ashary et al., 2018). 

The attachment and implantation of embryos can be strongly impacted by stress during the preimplantation phase. Stress, including any external stimulus that interferes with the embryo’s normal growth and development, can cause modifications to cell cycle progression, cell metabolism (Xie et al., 2011; Mansouri et al., 2012), and transcriptional processes (De Nadal et al., 2011). The different stressors can affect embryos both *in vivo* and *in vitro*. Stressors, including maternal disease, endocrine disruption, and toxins, can affect embryo development under physiological conditions (Feuer and Rinaudo, 2012). 

In contrast to natural conception, the implementation of assisted reproductive technology (ART) introduces stressors that may have an impact on the success of embryo implantation. The composition of the culture media, the techniques used during fertilization, and the amount of oxygen available during incubation are all important factors that affect the quality and potential for embryo implantation (Feuer and Rinaudo, 2012; Reshef et al., 2022). These factors can lead to increased production of reactive oxygen species (ROS) during the preimplantation phase. While biological concentrations of ROS are vital for key embryonic processes such as pronuclear formation and cell proliferation, elevated ROS levels can alter embryonic gene expression and increase DNA damage, which may lead to implantation failure (Deluao et al., 2022; Musson et al., 2022).

It has been reported that maternal hormones, including leptin, adrenaline, cortisol, and progesterone, contribute to stress by redirecting maternal and embryonic energy away from an ideal developmental process for the embryo. Different protein kinases regulate the response of embryos to stress by mediating different transcription factors, such as SAPK, p38MAPK, and PI3K (Puscheck et al., 2015). 

Recent reports implicate extracellular vesicles (EVs) as alternative mediators of signal transfer (Morales Dalanezi et al., 2019; Chan et al., 2020; Wu et al., 2021). EVs are nanoscale, membrane-bound vesicles released from cells into the surrounding environment. These particles perform vital functions in intercellular communication, including cell-to-cell signaling and the exchange and transfer of molecular cargo between cells. Previous studies have elucidated the bidirectional regulation of embryo-maternal communication through EVs (Evans et al., 2019). Several reports have highlighted the role of EV-mediated communication in the preparation of optimal conditions for successful implantation (Godakumara et al., 2022; Guzewska et al., 2023). EVs released by the embryo carry a cargo of bioactive molecules, including miRNAs, proteins, and lipids (Foster et al., 2016; Bazzan et al., 2021), which can modulate endometrial gene expression, promote changes in cellular signaling pathways, and ultimately enhance receptivity for implantation (Godakumara et al., 2021; Muhandiram et al., 2023). Conversely, the endometrium also secretes EVs that can impact trophoblast function and embryo development. These endometrium-derived EVs contain factors that regulate trophoblast adhesion, invasion, and differentiation, which are crucial processes for successful implantation. Additionally, they may carry signals that modulate immune responses at the maternal-fetal interface, contributing to the establishment and maintenance of pregnancy (Evans et al., 2019).

More importantly, EVs have been reported to facilitate the transfer of stress signals between different cells. For instance, follicular fluid from heat-stressed cows contains EVs that alter the gene expression of in vitro-matured oocytes (Morales Dalanezi et al., 2019). EV-mediated communication under stressed conditions, although less studied in embryo-maternal communication, is well documented in other research areas. Studies have reported that EVs derived from donor cells under oxidative stress exert distinct functions in recipient cells compared to those without stress. For instance, in cancer biology, EVs from stressed cells facilitate tumor progression and metastasis (Kucharzewska et al., 2013). EVs from cancer cells can either favorably regulate the host immune system, enabling it to identify and eradicate tumor cells, or conversely, cause immune suppression, which aids in the growth of cancer (Wu et al., 2021). In cardiovascular research, hypoxia-induced EVs contribute to cardiac remodeling and vascular repair by promoting angiogenesis (Belting and Christianson, 2015). According to research by Chan et al., spermatozoa cultured with EVs extracted from epididymal epithelial cells that had been subjected to stress resulted in offspring with altered neurodevelopment and adult stress reactivity (Chan et al., 2020).

Although EV communication in various reproductive processes has garnered significant attention, a significant gap persists in comprehending the mechanisms by which these nanosized particles facilitate the transmission of stress from maternal cells to the embryo. The effects of stress on EV cargo of endometrial cells and whether these modifications can cause particular stress reactions in trophoblast cells are still unknown. It is critical to comprehend this mechanism because stress-induced alterations in EV-mediated communication of the embryo and the endometrium could affect the implantation process. In our study, an *in vitro* model was utilized to simulate the preimplantation environment, employing the JAr human choriocarcinoma cell line to mimic trophoblast cells and the RL95-2 adenocarcinoma cell line to represent the mid-secretory endometrium (Devor et al., 2020; Ran et al., 2020; Godakumara et al., 2021). CoCl_2_, which mimics physiological hypoxic stress, was used to induce stress in endometrial cells (Muñoz-Sánchez and Chánez-Cárdenas, 2019; Xu et al., 2021). Based on this model, we investigated the influence of stress on the miRNA content of endometrial cell-derived EVs, as well as their consequential effect on gene expression in JAr spheroids, providing insights into the molecular mechanisms underlying stress-related disturbances in early embryo-maternal communication.

The objective of our study is to determine the extent to which EV miRNA contributes to the transcriptional changes observed in trophoblasts treated with these specific EVs. We hypothesize that stress in endometrial cells can change EV cargo, specifically miRNA, which can be transferred to the trophoblast and induce transcriptional changes. We utilized next-generation sequencing (NGS) to analyze EV miRNA and cellular mRNA, to detect transcriptomic changes in trophoblast cells and identify their potential causes by comparing the miRNA profiles and target gene expression across different experimental conditions. This study uniquely focuses on the impact of stress-induced changes in EV cargo with the expectation of observing oxidative stress in endometrial cells impacting EVs small RNAs which ultimately lead to transcriptional changes in the embryo and influencing signaling pathways potentially contributing to implantation failure and early onset of the epigenetic profile of the offspring.

## 2 Materials and methods

### 2.1 Experimental design

**FIGURE 1:**
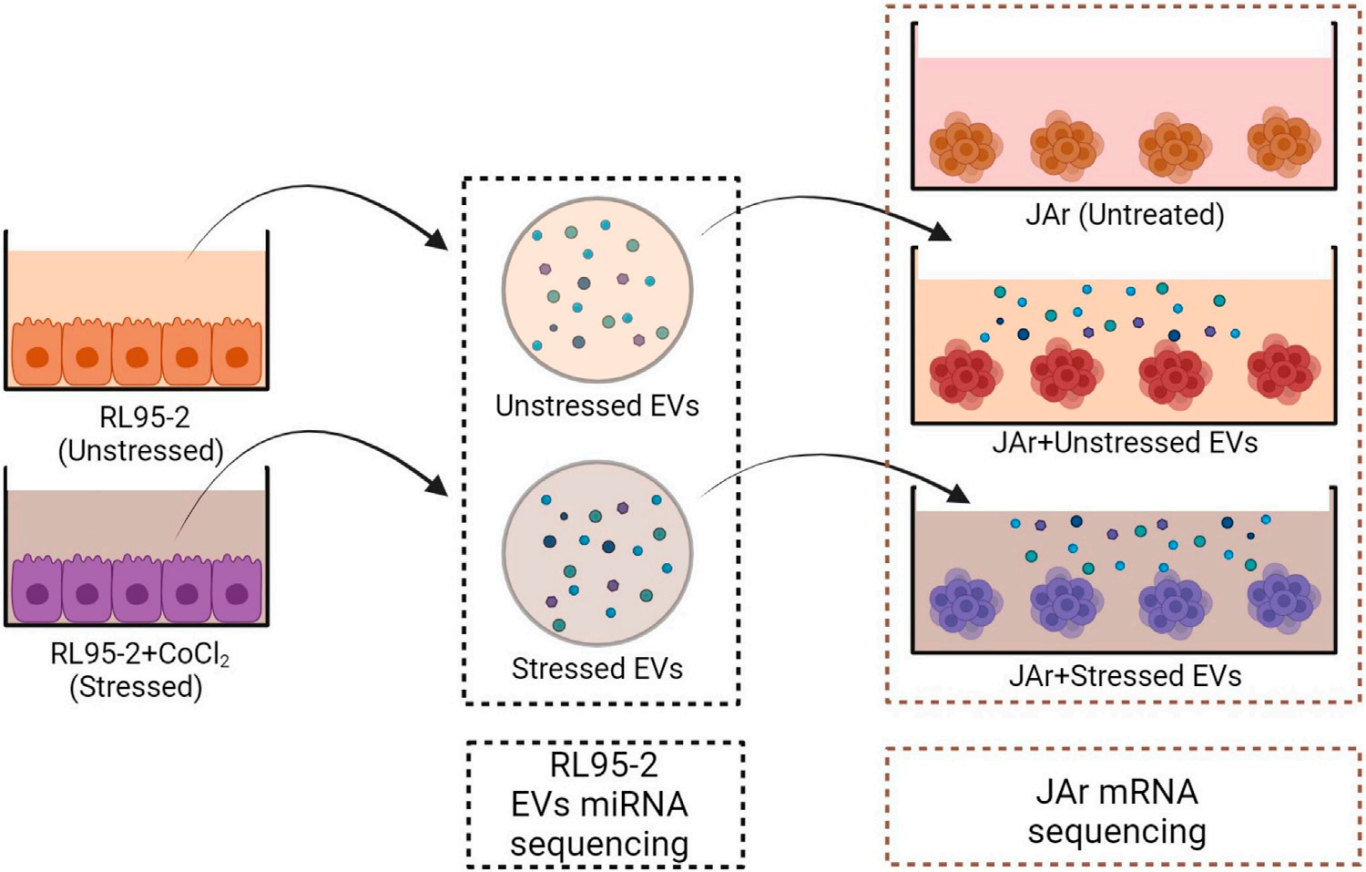
Schematic design of the experimental plan. EV isolation was performed from both stressed and unstressed RL95-2 cells. Multiple aliquots of EV samples were prepared. The miRNA profiles of the EV samples were evaluated using miRNA sequencing. JAr spheroids were treated with different EVs, and the transcription profile of JAr cells was investigated by mRNA sequencing.

The following experiments were performed with three experimental groups to compare the transcriptional profiles of JAr spheroids treated with EVs derived from stressed or unstressed RL95-2 cells and untreated JAr spheroids (Figure 1).

EV isolation was performed from two distinct groups of RL95-2 cells in the presence or absence of CoCl_2_. RL95-2 cells were cultured in T75 flasks until they reached 75% confluency. The cells were washed with Dulbecco’s phosphate-buffered saline without Ca2+ and Mg2+ (DPBS, Verviers, Belgium), and EV-depleted media was added to the cells with or without 250 µM CoCl_2_ (Sigma‒Aldrich). After incubation for 24 h, EVs were isolated from the conditioned media. The concentration of the isolated nanoparticles was measured using NTA. Moreover, cell viability and HIF1α expression were measured to confirm that the stress was induced in the RL95-2 cells without significant impact on cell viability. A cobalt assay kit was used to rule out the possibility of CoCl_2_ contamination in EV samples.

Afterwards, approximately 5,000 JAr spheroids made of 2.5 × 10^6^ cells were prepared based on the abovementioned protocol. Each group of spheroids was treated with 5 × 10^8^ EVs derived from stressed or unstressed RL95-2 cells for 24 h on a rotating gyratory shaker. Negative controls were prepared using supplemented media in place of EVs. After incubation, the JAr spheroids were collected and subjected to total RNA extraction. Additionally, RNA extraction was performed for similar aliquots of EVs which were used for supplementation.

Finally, the RNA samples from EVs and JAr spheroids were subjected to mRNA and miRNA sequencing. Bioinformatic analysis of RNA and miRNA sequencing data was performed to identify differentially expressed transcripts between the treated and control groups. Also, putative target prediction was performed for the detected miRNAs.

### 2.2 Cell culture and spheroid preparation

The human endometrial adenosquamous carcinoma cell line RL95-2 was purchased from American Type Culture Collection (ATCC CRL-1671, Teddington, United Kingdom). RL95-2 cells were routinely cultured in Dulbecco’s Modified Eagles medium F12 (DMEM 12-604F, Lonza, Verviers, Belgium) supplemented with 10% fetal bovine serum (FBS) (Gibco™, 10500064), 1% penicillin streptomycin (P/S) (Gibco™, 15140122, Bleiswijk, Netherlands), and 5 μg/mL insulin (human recombinant insulin, Gibco™, Invitrogen, Denmark) in 5% CO2 at 37°C.

The human choriocarcinoma cell line JAr (HTB-144™, Teddington, United Kingdom) was also obtained from ATCC. JAr cells were cultured in a T75 flask in RPMI 1640 media (Gibco, Scotland) supplemented with 10% FBS, 1% L-glutamine and 1% P/S at 5% CO2 at 37°C. After reaching 70% confluency, the JAr cells were washed with Dulbecco’s phosphate-buffered saline without Ca2+ or Mg2+ (DPBS, Verviers, Belgium). The cells were harvested using trypsin–EDTA (Gibco^®^ Trypsin, New York, United States) and pelleted by centrifugation at 250 × g for 5 min. The cells were cultured in 5 mL of supplemented RPMI 1640 medium in 60 mm Petri dishes at 5% CO2 at 37°C. The cells were kept on a gyratory shaker (Biosan PSU-2 T, Riga, Latvia) set at 320 rotations per min (rpm) for 24 h. The multicellular spheroids were used as preimplating embryo mimetics *in vitro*.

### 2.3 Cell viability analysis

A Live/dead^®^ viability/cytotoxicity assay kit (Molecular Probes, Eugene, Oregon, United States) was used to confirm the viability of the cells following the manufacturer’s instructions. In summary, working solutions of calcein AM (acetoxymethyl ester of calcein) and EthD-1 (ethidium homodimer-1) were prepared at final concentrations of 2 μM and 4 μM, respectively. Fluorescence microscopy was used to confirm the viability of the cells after the working solution was introduced directly onto the cells, following a 30-min incubation at room temperature.

### 2.4 Quantitative real-time PCR

The expression level of the gene of interest in RL-95-2 cells was verified using RT‒qPCR. Primers were designed using NCBI primer blast (Ye et al., 2012), and Integrated Genome Technologies-IDTTM (Owczarzy et al., 2008) was used to further assess primer quality (refer to Online Resource 1). Exon‒exon junction-spanning sequences were selected when primers were designed using Primer-BLAST. Primers were purchased from Microsynth AG in Wolfurt, Austria. Using FIREScript RT cDNA Synthesis mixTM with oligo (dT) and random primers (Solis BioDyne, Estonia), reverse transcription of RNA was performed with 200 ng of initial RNA. The evaluated gene transcripts were measured using on a QuantStudio 12K FlexTM real-time PCR system (Thermo Fisher Scientific) utilizing HOT FIREPol^®^ EvaGreen^®^ qPCR Supermix (Solis BioDyne, Estonia). The program for thermal cycling included enzyme activation at 95°C for 15 min; 40 cycles of denaturation at 95°C for 20 s; annealing at 60°C for 20 s; and extension at 72°C for 20 s. The specificity of PCR amplification was assessed using melting curve analysis. The fold change values obtained were analyzed using the 2^−ΔΔCT^ method (Winer et al., 1999). Beta-2-microglobulin (B2M) and staphylococcal nuclease and tudor domain containing 1 (SND1) were used for normalization. qPCR was performed in triplicate for each sample.

### 2.5 Preparation of EV-depleted medium

The ultrafiltration process, described by Kornilov et al., in 2018, was used to prepare EV-depleted FBS. In summary, Amicon Ultra15 centrifugal filters (100 kDa, Merck KGAA, Darmstadt, Germany) were used to filter the FBS at 3,000 × g for 55 min. This approach efficiently depletes EVs from FBS (Kornilov et al., 2018). To prepare the EV-depleted complete media, the filtered FBS was added as a 10% supplement to the complete culture media that were specifically prepared for the various cell types mentioned above.

### 2.6 EV isolation and characterization

The medium of the cells was changed to EV-depleted media when the cells were 70% confluent. After 24 h, the media were collected for EV isolation, initiating the process with sequential centrifugation in three steps: ×400 g, ×4,000 g and ×10,000 g for 10 min each at 4°C. The supernatant obtained from the preceding step was utilized for each subsequent centrifugation. Subsequently, the supernatant obtained after the final centrifugation step was concentrated to a final volume of 500 µL using Amicon^®^ Ultra15 centrifugal filters with a 10 kDa cutoff. EV isolation was performed by employing size exclusion chromatography (SEC) in a 10 cm column containing a cross-linked 4% agarose matrix of 90 μm beads (Sepharose 4 fast flow™, GE HealthCare Bio-Sciences AB, Uppsala, Sweden) (Midekessa et al., 2020). Fractions 7 to 10 (500 µL each) were collected using Amicon^®^ Ultra15 centrifugal filter devices (10 kDa cutoff). The size and concentration of the isolated EVs were quantified by nanoparticle tracking analysis (NTA) using ZetaView (Particle Metrix GmbH, Inning am Ammersee, Germany). The complete characterization of EVs from RL95-2 has been performed earlier according to the MISEV guidelines as described in our previous publication (Hart et al., 2023).

### 2.7 Cobalt colorimetric assay

The absence of CoCl_2_ residues in the purified EV samples was investigated using a cobalt colorimetric assay kit (catalog #K505-100) according to the manufacturer’s protocol. Briefly, six serial dilutions (0–50 nmol) of cobalt standard with an original concentration of 1 mM were prepared and adjusted to a final volume of 200 μL/well in a 96-well plate. Additionally, 15 μL of each EV sample was combined with distilled water to a final volume of 200 μL. Then, 10 μL of the cobalt reagent was added to each well containing the samples or the cobalt standard. After mixing well, the plate was incubated at room temperature for 10 min. Finally, the absorbance was measured colorimetrically (at 475 nm) with a 96-well plate reader. Then, a standard curve was derived, and the COCl_2_ residue concentrations in the test samples were calculated.

### 2.8 RNA extraction and quality control

TRIzol Reagent (Invitrogen) was used to extract total RNA from endometrial cell lines, trophoblast cell lines, and EVs. To enhance the effectiveness of RNA extraction, each sample’s lysis buffer was supplemented with 2 μL of UltraPureTM Glycogen (Cat. no. 10814–010, Thermo Fisher Scientific, Bleiswijk, Netherlands). The RNA precipitate was subjected to three consecutive washes with 70% ethanol to ensure purity. The quantitative and qualitative analysis of RNA was conducted by measuring the absorbance at 260 nm using a Nanodrop 2000 spectrophotometer (Thermo Scientific), while integrity assessment was performed using an Agilent 2100 Bioanalyzer.

### 2.9 mRNA library preparation and sequencing

The VAHTS universal V8 RNA-seq Library Prep Kit for Illumina NR605 was used to construct the mRNA library and strand-specific mRNA library in strict accordance with the protocol provided by the manufacturer (Vazyme Biotech, Nangjing, PRC). The library preparations were sequenced on the Illumina NovaSeq 6000 platform (Illumina, San Diego, CA), and reads were generated using the NovaSeq 6000 S4 Reagent Kit (Illumina, San Diego, CA).

### 2.10 Small RNA library preparation and sequencing

A total of 0.5 μg of RNA per sample was used as input material for the RNA sample preparations. Sequencing libraries were generated using the NEBNext Ultra small RNA Sample Library Prep Kit for Illumina (NEB, United States) following the manufacturer’s recommendations, and index codes were added to attribute sequences to each sample. Library quality was assessed on an Agilent Bioanalyzer 2100 system. Clustering of the index-coded samples was performed on a cBot Cluster Generation System using the TruSeq PE Cluster Kit v4-cBot-HS (Illumina) according to the manufacturer’s instructions. After cluster generation, the library preparations were sequenced on an Illumina NovaSeq 6000 platform (Illumina, San Diego, CA), and paired-end reads were generated.

### 2.11 Processing, alignment, and quantification of RNA sequencing (RNAseq) reads

With FASTQC v 0.12.0, the raw read quality was evaluated (Brown et al., 2017). Trimmomatic v0.39 was used to trim and remove adaptor sequences (Bolger et al., 2014). The human reference genome, hg38, was used to align the mRNA reads. HISAT2 (Kim et al., 2019) was applied for the alignment, with the default parameters. Using the Ensembl *H. sapiens* annotation file (GRCh38.97) and miRbase annotation file (Griffiths-Jones, 2004; Kozomara et al., 2019) for respective feature annotations, featureCounts (Liao et al., 2014) with default parameters was used to derive exon-level read counts. To conduct further tests for differential expression, genes/miRNAs that had at least 10 reads across all samples in at least one of the experimental groups were retained for analysis.

### 2.12 Differential gene expression analysis

Using edgeR (Bioconductor version: 2.7), differential expression (DE) analysis was performed in R version 4.3.2 (Robinson et al., 2010). Based on the trended dispersions, tagwise dispersion estimates were calculated. Statistical comparisons were carried out with a generalized linear model and likelihood ratio tests, which also considered the experimental batch. The False Discovery Rate (FDR) was calculated using the Benjamini-Hochberg method. This approach provides a more accurate interpretation of the data, enhancing the credibility of the study’s conclusions. Genes with a FDR of less than 0.05 were considered to have significantly altered expression.

The ReactomePA program and Reactome Pathway database annotations (Yu and He, 2016) were used to perform gene set enrichment analysis (GSEA) and pathway overrepresentation analysis. Full gene lists from DE analysis were ranked by −log10p × log2FC, where FC is the fold-change and p stands for unadjusted *p* values. GSEA was utilized for these gene lists. The ggplot2 tool was utilized to show the principal components, which were computed using the prcomp function from the Stats package (Wickham and Wickham, 2016). Heatmap rendering with hierarchical clustering based on Euclidean distance was accomplished using the pheatmap package (Kolde, 2012).

### 2.13 Prediction of potential targets of RL95-2 specific miRNAs in JAr cells

We sourced the entire list of anticipated target transcripts from the miRDB (Chen and Wang, 2020). Only targets with a high confidence score (target score of ≥ 90) were retained after filtering. The list of expected miR targets at the gene level was obtained by converting the REFSEQ transcript IDs to ENSEMBL gene IDs using the R package AnnotationDbi (Nie et al., 2009). Matching the ENSEMBL IDs was performed to identify potential miRNA targets in the JAr gene expression dataset.

### 2.14 Statistical analysis and visualization

All experiments were performed with three independent replicates. The normal distribution of the data was confirmed by the Kolmogorov–Smirnov test. Statistical analysis of differences in cell viability and the CoCL_2_ concentration between the two groups was carried out using independent sample t tests with Graph-Pad Prism 7 software (Graph-Pad Software Inc., San Diego, California). The data generated are expressed as the mean ± standard deviation (SD).

## 3 Results

### 3.1 Hypoxia was induced by CoCl_2_ in RL95-2 cells without any significant effects on cell viability

**FIGURE 2:**
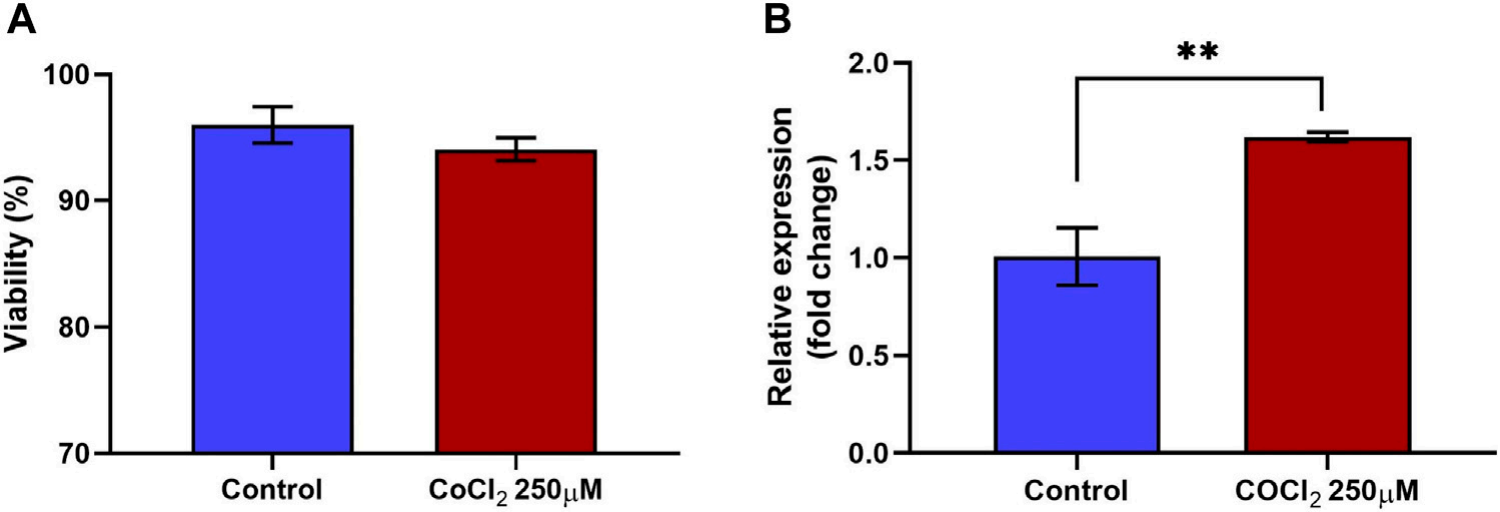
Viability and relative expression of HIF1A in RL95-2 cells in response to CoCl_2_. RL95-2 cells were treated with 250 µM CoCl_2_ to induce hypoxic stress. The viability of the stressed cells was measured using a live/dead staining protocol, and the gene expression of the stress marker HIF1A in the treated cells was measured to confirm the stressed condition **(A)** The viability percentage of RL95-2 cells treated with CoCl_2_. There were no significant differences in viability Between treated and untreated cells **(B)** The relative expression of the hypoxic stress marker HIF1A in CoCl_2_-treated RL95-2 cells compared to that in untreated control cells was measured. In cells treated with CoCl_2_, there was a significant upregulation of HIF1A compared to the untreated cells. Values are presented as mean ± SD. **, *p* < 0.01.

Treatment of RL95-2 cells with CoCl_2_ did not significantly reduce cell viability. The percentages of viable cells in the control and treated groups were 96% ± 1.45% and 94% ± 0.90%, respectively. Three independent biological replicates were performed to ensure the consistency of the results (n = 3). Despite a slight reduction in cell viability observed with 250 μM CoCl_2_, these differences did not reach statistical significance compared to those of the control group, as shown in Figure 2A.

Furthermore, 250 µM CoCl_2_ was adequate to induce oxidative stress in RL95-2 cells, as confirmed by the gene expression of hypoxia-inducible factor 1 subunit alpha (HIF1A). Compared to the control group, the expression of HIF1A in the treated group was significantly (*p* < 0.05) upregulated, with a fold change of 1.62 (Figure 2B). Therefore, the same concentration of CoCl_2_ was used for subsequent experiments. Furthermore, these findings confirmed that although the cells remained viable, stress was induced, as indicated by the upregulation of the transcription factor HIF1α, which is a marker of oxidative stress.

The preliminary experiments included a range of CoCl_2_ concentrations (200 μM, 250 μM, and 300 µM). The other concentrations of CoCl_2_ were tested to determine the optimal level that induces stress without significantly affecting cell viability. 250 μM of CoCl_2_ was chosen based on viability assays and stress marker analysis, which indicated that lower concentrations did not induce significant stress response, while higher concentrations (300 µM) led to excessive cell death. These data are presented in Supplementary Figures S1A, S1B.

### 3.2 EV samples were not contaminated with residual cobalt

The cobalt colorimetric assay data revealed that the concentrations measured in both stressed and unstressed RL95-2 cell-derived EV samples were below the detection level of the assay.

### 3.3 JAr spheroids treated with EVs derived from unstressed and stressed RL95-2 cells exhibited different transcription profiles

**FIGURE 3:**
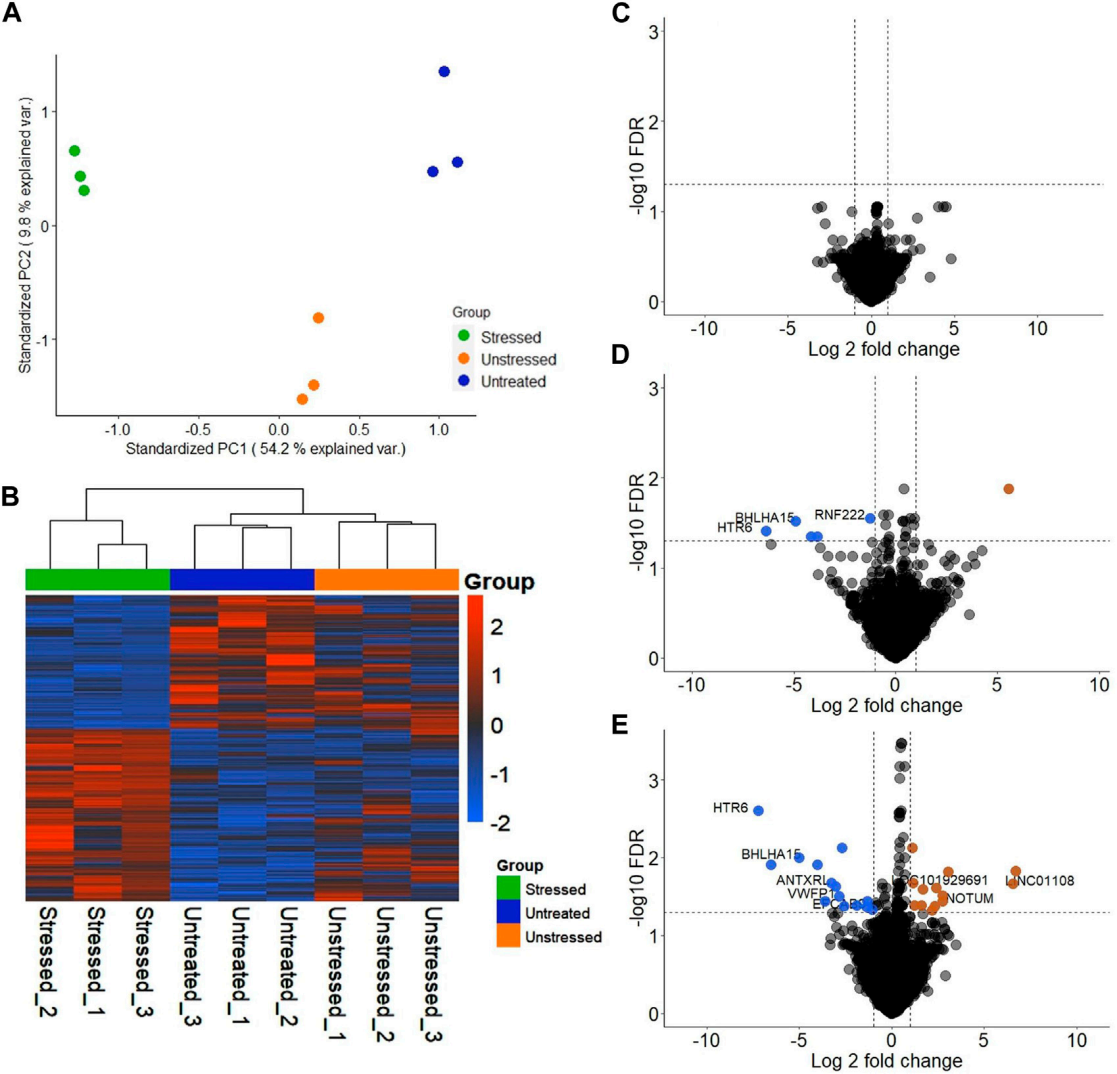
Transcriptional profile of JAr spheroids treated with EVs derived from unstressed and stressed RL95-2 cells compared to those in the untreated group **(A)** Principal component analysis (PCA) demonstrating the two-dimensional distribution of all the genes that were differentially expressed among the three groups **(B)** Heatmap illustration of differentially expressed genes (DEGs) in the three groups: JAr spheroids with stressed RL95-2EVs, JAr spheroids with unstressed RL95-2EVs and JAr spheroids without treatment **(C)** Volcano plot shows that there were no transcripts that were differentially expressed between JAr spheroids treated with unstressed RL95-2EVs and untreated group **(D)** The volcano plot illustrates the comparison of genes showing significant differential expression between JAr spheroids treated with EVs from stressed versus unstressed RL95-2 cells, with enriched genes marked in orange and depleted genes marked in blue **(E)** This comparison plot highlights differentially expressed transcripts between JAr spheroids treated with EVs from stressed RL95-2 and those from the untreated group, with enriched or depleted genes highlighted in orange or blue, respectively.

Significant differences were observed in the transcriptomic profiles of JAr spheroids treated with stressed and unstressed RL95-2 cell-derived EVs. According to the PCA plot, there was considerable variation between the three treatment groups, while the intragroup variations were small (Figure 3A). The heatmap shows large differences in the mRNA expression profile between the stressed group and the other two groups (Figure 3B).

In differential gene expression analysis, there were no genes with significant differential expression in the unstressed EV-treated group compared to the untreated group (Figure 3C). Additionally, 5 significantly downregulated genes and 1 significantly upregulated gene were detected in the JAr spheroids treated with stressed RL95-2EVs compared to the JAr spheroids treated with unstressed RL95-2EVs (Figure 3D). Compared to the untreated group, 27 transcripts in the stressed-EV-treated group exhibited significantly altered expression, 14 of which were downregulated and 13 of which were upregulated (Figure 3E).

### 3.4 Pathways related to cell apoptosis, collagen degradation, selenocysteine synthesis, and metabolism were significantly enriched in JAr spheroids treated with stressed EVs

**FIGURE 4:**
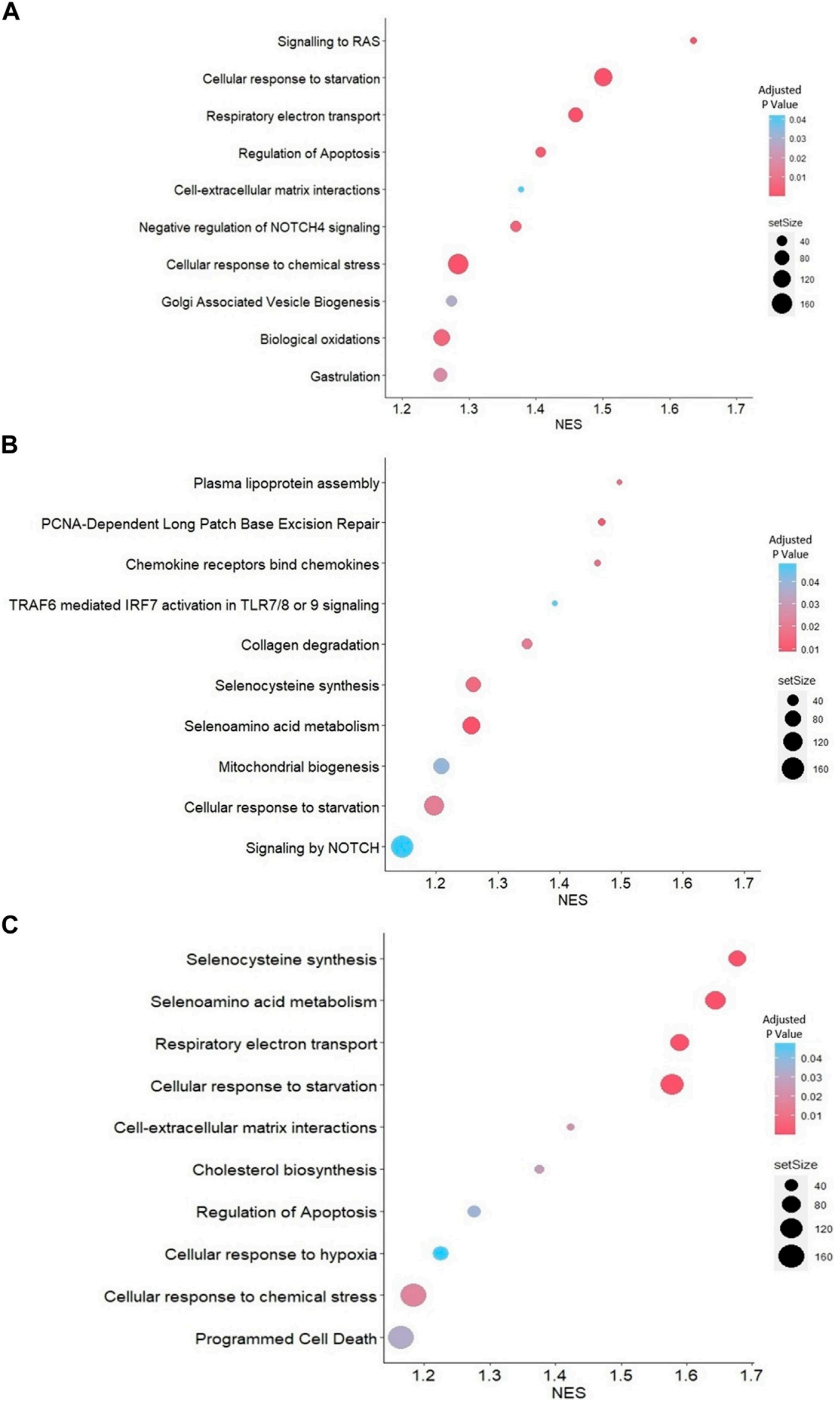
GSEA with Reactome pathways **(A)** Highly represented pathways associated with the genes which were differentially expressed between JAr spheroids treated with unstressed RL95-2EVs and untreated JAr spheroids **(B)** Pathways which are overrepresented by DEGs in JAr spheroids treated with EVs from stressed RL95-2 cells compared to unstressed RL95-2 cells **(C)** Overrepresented pathways by differentially expressed genes in JAr spheroids treated with stressed RL95-2EVs compared to untreated JAr spheroids. Pathways were arranged according to the normalized enrichment score (NES).

Reactome-based GSEA was performed for the DEGs between each group. The analysis demonstrated that pathways related to gastrulation, negative regulation of *NOTCH4* signaling, and cell-extracellular matrix interactions were activated in JAr spheroids treated with unstressed RL95-2EVs compared to untreated JAr spheroids (Figure 4A). Among the pathways highly represented by DEGs in JAr spheroids treated with stressed RL95-2EVs compared to unstressed RL95-2EVs, signaling by *NOTCH*, mitochondrial biogenesis, collagen degradation, selenocysteine synthesis and metabolism were highly relevant to the studied biological system (Figure 4B). Interestingly, programmed cell death and apoptosis, cellular response to hypoxia and chemical stress, as well as selenocysteine synthesis and metabolism pathways are among the highly represented pathways associated with the response of JAr spheroids to EVs from stressed RL95-2 compared to the untreated group (Figure 4C).

### 3.5 Different profiles of miRNAs were discovered in EVs derived from stressed RL95-2 compared to the unstressed RL95-2 cells

**FIGURE 5:**
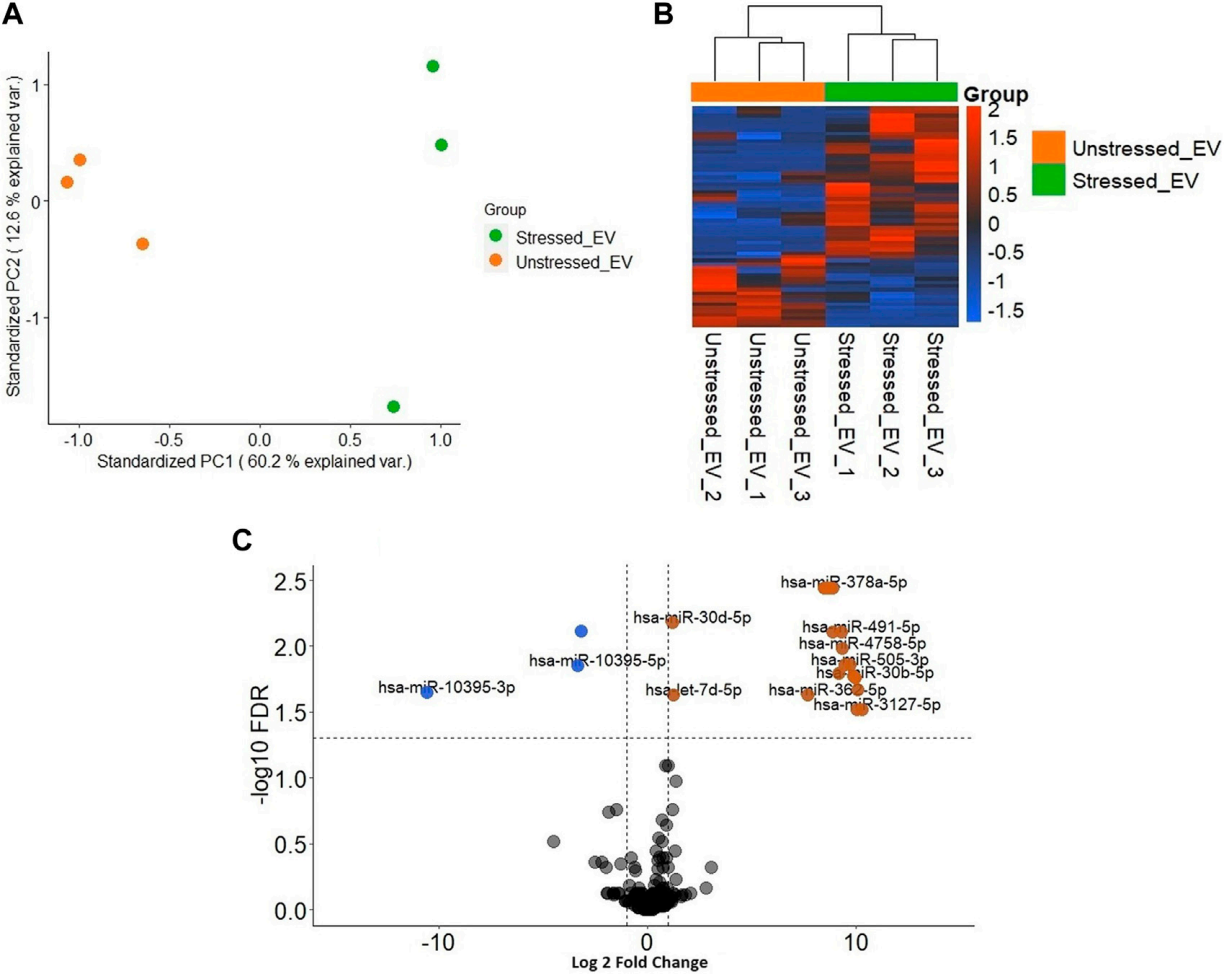
Different distribution of miRNAs in EVs derived from stressed and unstressed RL95-2 cells **(A)** Principal component analysis (PCA) shows the overall separation of differentially expressed miRNAs between stressed and unstressed EVs in a two-dimensional distribution **(B)** Heatmap of significant differentially expressed miRNA in unstressed and stressed EVs **(C)** Volcano plot of the miRNAs with significantly different expression levels. miRNAs that were significantly enriched or depleted in the stressed EVs compared to the unstressed EVs are highlighted in orange and blue, respectively.

Significantly distinct populations of miRNAs were detected between stressed and unstressed samples. The PCA plot demonstrated greater intragroup variation in the stressed samples than in the unstressed samples; however, the segregation of the two groups was more dominant (Figure 5A). The heatmap illustrates the general variation in transcripts between the two distinct groups (Figure 5B). In stressed EVs compared to unstressed EVs, significant changes (FDR ≤ 0.05) in miRNA expression were detected. Specifically, the volcano plot shows that three miRNAs were depleted (logFC <−1), while 22 miRNAs were enriched (logFC > 1) (Figure 5C). These results suggest that the induction of oxidative stress in RL95-2 cells can significantly alter the miRNA profile of EVs.

### 3.6 GSEA of RL95-2EV-targeted genes in JAr spheroids reveals significant enrichment of cellular response to stress and stimuli pathways

**FIGURE 6:**
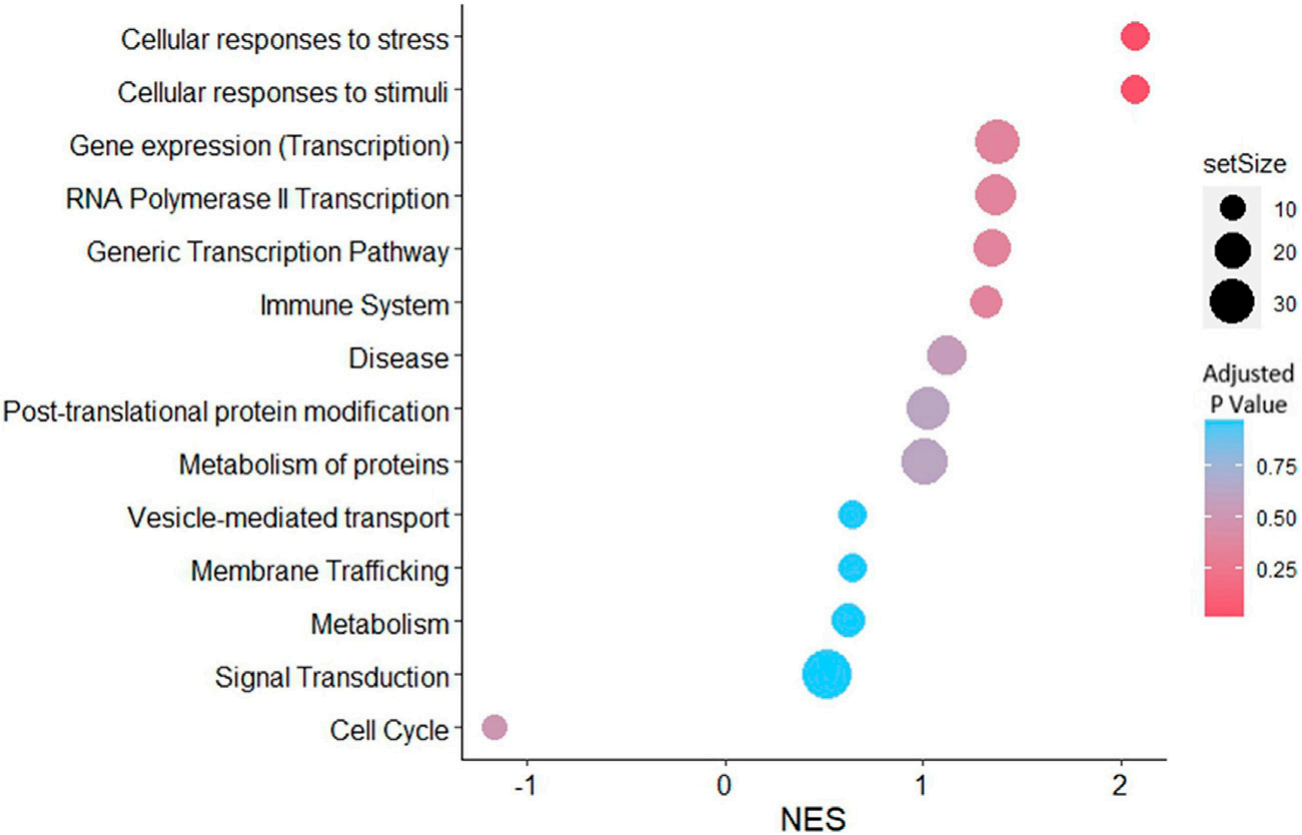
GSEA with reactome pathways illustrates the significant enrichment of pathways related to cellular responses to stress and cellular responses to stimuli, associated with genes that were differentially expressed in stressed EVs compared to unstressed EVs. Pathways were arranged according to the normalized enrichment score (NES).

Gene set enrichment analysis was performed for the predicted targets of the detected miRNAs in the EV samples. According to the network analysis, 14 pathways were enriched in the JAr spheroids treated with stressed EVs compared to the unstressed EV-treated group (Figure 6). However, only two pathways, cellular responses to stress (R-HSA-2262752) and cellular responses to stimuli (R-HSA-8953897), were significantly enriched.

## 4 Discussion

Stress is recognized as a significant obstacle to the establishment of a successful implantation (Chen et al., 2016; Zhou et al., 2019). However, the underlying mechanism of stress transfer between mothers and embryos remains yet to be elucidated. While, a number of studies have explored the role of EVs derived from follicular fluid, oviducts, and the endometrium (Evans et al., 2019), in embryo-maternal communication the specific involvement of EV-mediated signaling between the embryo and the endometrium under stressed conditions remains relatively understudied. The present study was designed to investigate the effect of oxidative stress in endometrial cells on the miRNA content of EVs and the impact of these stressed EVs on the gene expression profile of JAr spheroids.

To ensure the validity of our experimental model, RL95-2 and JAr cell lines were both authenticated by ATCC using Short Tandem Repeat (STR) profiling before conducting experiments. This step confirms the identity and purity of the cell lines. Figures 3C–E demonstrated the genes with significant changes, however, housekeeping genes including B2M, 18S, and ACTB were not significantly altered. This data indicated that different experimental conditions did not affect the fundamental characteristics of the cell lines while influencing particular genes which are mainly related to the early stages of embryo-maternal cross-talk. Moreover, common markers of trophoblast for instance, cytokeratin 7 (KRT7), and human leukocyte antigen G (HLA-G) which play significant roles in the epithelial cell differentiation, and immunological functions of trophoblast cells, were not significantly changed.

Interestingly, our results revealed significant downregulation of 5-hydroxytryptamine receptor 6 (*HTR6*) and basic helix-loop-helix family member a15 (*BHLHA15*) in the JAr spheroids when treated with stressed EVs compared to unstressed EVs and untreated groups (Figures 3D, E). 5-Hydroxytryptamine (5-HT), also known as serotonin, controls the release of reproductive hormones and shields the placenta from cellular death (Hadden et al., 2017). Also, the expression of basic helix-loop-helix (*BHLH*) transcription factors has a significant impact on the development of mouse trophoblasts, coordinating vital events necessary for healthy placental growth and function (Meinhardt et al., 2005). Therefore, our results suggested that stressed EVs negatively impact trophoblasts by reducing the expression of *BHLHA15* and *HTR6*.

Our expectation was to observe endometrial-derived EVs altering the transcription of specific genes which regulates pathways related to early embryo development and implantation. Consistent with previous research, our data demonstrated that unstressed EVs trigger specific pathways to support early embryo development and implantation. These pathways included gastrulation, negative regulation of NOTCH4 signaling, and cell-extracellular matrix (ECM) interaction (Figure 4A). An *in vitro* model study conducted in bovines revealed that oviductal EVs contain specific microRNAs that downregulate the expression of genes linked to the gastrulation process (Bauersachs et al., 2020). Furthermore, different studies have highlighted the role of *NOTCH* signaling in embryo implantation (Rombauts et al., 2014). For instance, it has been reported that *NOTCH4* expression is reduced during cytotrophoblast differentiation (Hunkapiller et al., 2011). Furthermore, we previously reported that RL95-2 cells treated with JAr EVs exhibit high expression of genes linked to ECM organization, which is known to have a functional effect on endometrial receptivity (Godakumara et al., 2021). These findings imply that endometrial EVs possess the capacity to deliver signals to support the successful implantation of embryos.

Conversely, stressed EVs negatively affected the implantation process through *NOTCH* signaling, mitochondrial biogenesis, and collagen degradation (Figure 4B). Fetal-maternal communication during implantation and placentation crucially relies on *NOTCH* signaling, while aberrant *NOTCH* expression is linked to preeclampsia and poor placentation (Rombauts et al., 2014). One of the critical regulators of oxidative stress is the *NOTCH* signaling pathway. In rat models, myocardial protection is conferred through crosstalk between *NOTCH1* and *NRF2*, leading to attenuation of ROS production (Zhou et al., 2020). Additionally, due to the impairment of mitochondrial function and induction of mitophagy by hypoxia (Xu et al., 2021), trophoblast cells seem to respond by upregulating mitochondrial biogenesis as a compensatory mechanism. Moreover, stressed endometrial EVs may contribute to implantation failure by inducing the collagen degradation pathway in trophoblast cells. Collagen is one of the key elements in the mammalian extracellular matrix. The expression of different varieties of collagen within trophoblasts has been documented in numerous investigations (Shi et al., 2020). Also, Xu et al. (2001) reported that trophoblast adhesion was more pronounced in the presence of collagen type I and collagen type IV than in the presence of other components of the extracellular matrix. Consequently, collagen degradation in trophoblast cells may impede the implantation process.

Interestingly, EVs released during oxidative stress exhibit dual functions, with both advantageous and detrimental effects. While they transport antioxidant molecules that assist in regulating the oxidative stress response within target cells, they also transport oxidized lipids and proteins, potentially leading to harmful effects (Yarana and St. Clair, 2017; Benedikter et al., 2018).The expression of *COX4-1*, a subunit of the respiratory chain, is significantly decreased in carcinoma cells treated with CoCl_2_ (Hervouet et al., 2008). Several studies have demonstrated that oxidative stress caused by CoCl_2_ can activate cell apoptosis through ROS production (Zou et al., 2001). An increase in ROS levels of the cells leads to the activation of antioxidant reactions and ROS-related signaling pathways (Hogan and Perkins, 2022). Selenocysteine, a vital element of selenoproteins, is pivotal in these biological processes. 

It has been reported that maternal supplementation with organic selenium during pregnancy resulted in increased levels of antioxidant capacities in maternal, placental, and fetal tissues, while decreasing inflammatory factors (Mou et al., 2020). Moreover, downregulation of SECISBP2, as selenocysteine incorporation factor, inhibit proliferation and invasion of human trophoblast cells by suppressing Akt and ERK signaling pathway (Li et al., 2017). Our results suggest that stressed EVs can disrupt antioxidant reaction of trophoblast cells by affecting selenocysteine synthesis and metabolism.

Another expected observation was the presence of distinct miRNA cargo within EVs derived from RL95-2 cells under stress conditions (Figures 5A, B). Previous studies have demonstrated that oxidative stress induces changes in EV cargo, leading to subsequent biological effects (Jiang et al., 2022). Salomon et al. reported that EVs from cytotrophoblasts subjected to oxidative stress carry distinct protein cargo, enhancing signals associated with cellular invasion and migration (Salomon et al., 2013). We fulfilled our aim to identify the contribution of EV miRNA in the transcriptional changes observed in trophoblasts treated with stressed EVs, by comparing possible miRNA targets with altered genes in trophoblast cells across different groups, Our results suggested that less than 10% of downregulated genes in JAr spheroids treated with stressed EVs versus unstressed EVs can be attributed to the miRNA-induced silencing process.

GSEA of the predicted targets of the differentially expressed miRNAs in the JAr spheroids revealed that the significantly enriched pathways were related to cellular responses to stress and stimuli (Figure 6). In the current study, we investigated the miRNA content of stressed and unstressed EVs to elucidate the underlying mechanisms governing the transcriptional profile of JAr spheroids. This analysis endeavours to unveil the influence of EV cargo on gene expression, with a particular emphasis on miRNAs as potential mediators of transcriptional alterations. The regulation of numerous crucial pathways involved in cellular adaptation to hypoxia may be facilitated by several miRNAs associated with stressed EVs (Bister et al., 2020). These findings suggest that EV miRNAs from stressed endometrial cells regulate the trophoblast to deal with oxidative stress.

This study was limited to investigating the effects of CoCl_2_-induced oxidative stress using an *in vitro* model. The *in vitro* environment, while useful for controlled studies, lacks the complexity and dynamic nature of *in vivo* systems. However, the implantation model using JAr and RL95-2 cells has been well-established since the 1970s (Rohde and Carson, 1993). We suggest that future research should incorporate more advanced models such as organoid systems including for instance both stromal and epithelial cells that better mimic endometrial tissues. Using animal models can also provide valuable insights into the transfer of stress via EVs between different cell types, particularly in embryo-maternal communication. These approaches would help further understanding of the physiological relevance of EV-mediated stress signaling in the context of pregnancy.

Our findings demonstrated that EVs derived from stressed cells significantly alter the response of recipient cells, which is partially caused by the miRNA cargo of the EVs. It is imperative to note that the EV samples were thoroughly devoid of any remaining CoCl_2_, thus confirming that the observed effects were solely attributable to the stressed EVs.

In conclusion, our results offer further validation for the proposition that EVs can elicit varied physiological responses in target cells, contingent upon the stress levels present in the originating donor cells. Furthermore, compared with unstressed EVs, stressed EVs can alter gene expression in trophoblast cells, which seems to be partially regulated by EV miRNAs. While unstressed EVs can modulate specific pathways to support successful implantation, stressed EVs have the potential to activate the apoptosis process in trophoblasts. JAr spheroids are observed to be engaging in cellular stress response pathways and facilitating mitochondrial biogenesis as a mean to counteract the detrimental effects of hypoxia. Additional research is directed toward elucidating the role of EVs originating from stressed tissues such as the uterus, fallopian tubes, placenta, and embryo itself in mediating intercellular signaling within reproductive organs. These findings could provide valuable insights into the mechanisms underlying reproductive dysfunction in stressed environments. Understanding the mechanisms underlying the use of EVs as mediators of stress transfer could ultimately guide the development of interventions aimed at managing infertility, and unexplained implantation failures that might be associated with stress.

## Data Availability

The datasets presented in this study can be found in online repositories. The names of the repository/repositories and accession number(s) can be found below: https://www.ncbi.nlm.nih.gov/, PRJNA1107387.
