# Supplementary material for: Extracellular vesicles as mediators of stress response in embryo-maternal communication
Source: Front Cell Dev Biol. 2024 Aug 5;12:1440849. doi: 10.3389/fcell.2024.1440849 (PMC11330882; doi:10.3389/fcell.2024.1440849)
Supplement: Supplementary file 1 [file DataSheet1.pdf]

Supplementary Material

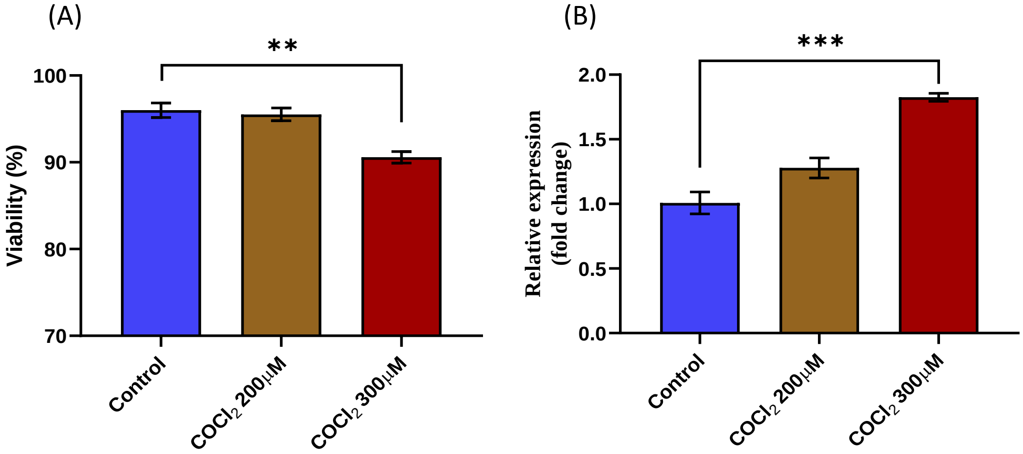

Supplementary Figure 1: Viability and relative expression of HIF1A in RL95-2 cells in response to different concentrations of $CoCl_2$. **(A)** The viability percentage of RL95-2 cells treated with $CoCl_2$. There was only a significant reduction in cell viability in the group treated with 300 µM of $CoCl_2$ compared to the control **(B)** The relative expression of the hypoxic stress marker HIF1A in $CoCl_2$-treated RL95-2 cells compared to that in untreated control cells was measured. The significant upregulation of HIF1A was observed only in the cells treated with 300 µM $CoCl_2$, compared to the untreated cells. Values are presented as mean±SD. **, $p < 0.01$ and ***, $p < 0.01$.
